# Switch to low-fat diet improves outcome of acute lymphoblastic leukemia in obese mice

**DOI:** 10.1186/s40170-018-0189-0

**Published:** 2018-11-01

**Authors:** Jonathan Tucci, Waseem Alhushki, Ting Chen, Xia Sheng, Yong-Mi Kim, Steven D. Mittelman

**Affiliations:** 1 Diabetes and Obesity Program, Center for Endocrinology, Diabetes and Metabolism, Children’s Hospital Los Angeles, Keck School of Medicine, University of Southern California, Los Angeles, CA USA; 2 Children’s Center for Cancer and Blood Diseases, Children’s Hospital Los Angeles, Keck School of Medicine, University of Southern California, Los Angeles, CA USA; 3 Present Address: Cure 4 The Kids Foundation, Las Vegas, NV USA; 4 Division of Pediatric Endocrinology, UCLA Children’s Discovery and Innovation Institute, David Geffen School of Medicine UCLA, 10833 Le Conte Ave, Los Angeles, CA 90095-1752 USA; 5 Present Address: Aptose Biosciences, San Diego, CA USA

**Keywords:** Obesity, Adipose tissue, Dietary intervention, Chemotherapy, Caloric restriction

## Abstract

**Background:**

It is becoming increasingly recognized that weight and nutritional status can impact cancer survival. We have previously shown that obese mice with syngeneic acute lymphoblastic leukemia (ALL) have poorer response to chemotherapy treatment than control mice. We therefore investigated whether dietary intervention could improve outcome from the most common pediatric cancer, ALL.

**Methods:**

Diet-induced obese (DIO) mice raised on a 60% calories from fat diet and control mice were implanted with syngeneic ALL cells. Some DIO mice were switched to the low-fat control diet. Survival from ALL was assessed without or with chemotherapy treatment starting at the time of the diet switch. Cells from DIO mice before and after diet switch were assessed by FACS for BrdU incorporation and phosphorylation status of AKT, S6K, and EIF2a. Similar experiments were done with human ALL xenografts. Mouse and human ALL cells were cultured in media with 10% or 5% fetal bovine serum, and sensitivity to chemotherapies assessed.

**Results:**

DIO mice had poorer survival (17%) after vincristine monotherapy than control mice on a 10% low fat diet (42%; *n* = 12/group; *p* = 0.09, log rank). However, switching obese mice to the low-fat diet prior to initiation of vincristine led to dramatically improved survival (92%, *p* < 0.01 vs both other groups). In vitro, FBS restriction made murine and human ALL cells more sensitive to vincristine. Interestingly, while serum restriction enhanced ALL sensitivity to dexamethasone and l-asparaginase, dietary switch did not improve survival of DIO mice treated with either drug in monotherapy. Thus, it appears that dietary intervention has a unique effect to improve ALL cell sensitivity to vincristine in vivo.

**Conclusions:**

We report herein that a dietary intervention can improve ALL outcome in a preclinical model. Further work is needed to identify the mechanisms of this effect and investigate potential impact on human leukemia in patients.

**Electronic supplementary material:**

The online version of this article (10.1186/s40170-018-0189-0) contains supplementary material, which is available to authorized users.

## Background

Obesity increases the incidence of many cancer types, and obese cancer patients have a higher risk of mortality from their disease [1]. In 2007, a retrospective review of two large cohorts demonstrated that obesity at the time of diagnosis increases risk of relapse in children with National Cancer Institute/Rome High-Risk acute lymphoblastic leukemia (HR-ALL) by 50% [2], a finding confirmed in meta-analysis [3]. In a separate cohort, we reported that obese children were 2.74 times more likely to be minimal residual disease (MRD) positive [4], which portends increased relapse risk and reduced event-free survival [5]. Moreover, recent studies show 1 in 3 children with ALL were overweight or obese at diagnosis [6, 7].

Much attention has been given to the role of diet on cancer outcomes. Meta-analysis has shown that cancer survivors who maintain high quality diets have lower overall mortality, though no significant reduction in cancer recurrence [8]. Less clear is whether dietary intervention during cancer treatment might improve outcome. Caloric restriction has garnered much attention in this realm, as it can potently reduce insulin-like growth factor 1 (IGF-1), a stimulator of cellular metabolism through activation of the PI3K/Akt/mTOR axis [9, 10]. Non-transformed cells respond to this low nutrient state by downregulating mTOR activity and entering a state of quiescence, which can protect them from some chemotherapies; however, cancer cells often exhibit constitutive activation of PI3K/Akt/mTOR [11]. This constitutive activation cannot be modulated by nutrient restriction, leading to retained or increased chemosensitivity of cancer cells [12, 13]. Additional mechanisms also likely contribute to the beneficial effects of caloric restriction, including reduced fuel availability, reduction of inflammation, and lower oxidative stress [9, 14].

We have developed a mouse model which recapitulates the clinical observation that obese children have worse ALL outcome; high-fat diet-induced obese (DIO) mice implanted with syngeneic ALL had a poorer survival outcome after treatment with either vincristine (VCR) or l-asparaginase [15, 16]. However, whether the effects of obesity on ALL outcome in mice or patients is reversible remains unknown. While caloric restriction has been shown to improve chemotherapy efficacy in mouse models of solid tumors [12], it has not been tested in the most common childhood cancer, acute lymphoblastic leukemia. Therefore, we designed the present study to test whether a dietary intervention could improve ALL outcome in obese mice.

## Methods

### Cell culture

Murine pre-B ALL cells were previously isolated from a BCR/ABL transgenic mouse (“8093 cells” [17]) and transduced with GFP. Human leukemia cell lines included BV173 (pre B Ph + ALL, ATCC) and Nalm-6 (B cell precursor leukemia, ATCC). Cells were authenticated by the University of Arizona Genetics core in November 2016 and tested negative for mycoplasma. Eight thousand ninety-three cells were cultured in McCoy’s 5A media (Invitrogen), supplemented with 1 mM sodium pyruvate, 2 mM Glutamax, 10 μg/mL gentamycin and either 5% or 10% FBS (Denville Scientific or Omega Scientific). Human cell lines were cultured in RPMI 1640 (Invitrogen), supplemented as above. Chemotherapy sensitivity experiments were performed over 72 h, with surviving viable cells counted by blinded observers in triplicate using trypan blue exclusion manually, or with a Countess II (ThermoFisher). LAX7 cells are patient-derived ALL cells with normal karyotype which were expanded by passage through NSG mice (mice described below) and used for xenograft [18]. Cells were cultured on OP-9 stroma cells with αMEM supplemented with 20% FBS, 100 IU/mL penicillin, and 100 μg/mL streptomycin.

### Mouse models

High fat diet-induced obese (DIO) and control C57Bl/6J mice were purchased from Jackson Laboratories (Bar Harbor, MI, USA). Mice had been raised on either a 60% (obese) or 10% (control) calories from fat diet (Research Diets, D12492 and D12450B, respectively). Because female mice do not become as significantly obese in this model and are not available as DIO mice from Jackson Laboratories, only male mice were used for these studies. Male NSG mice (NOD.Cg-*Prkdc*^*scid*^ *Il2rg*^*tm1Wjl*^/SzJ) are NOD/SCID IL2-receptor gamma chain knockout mice commonly used for xenograft. NSG mice were made obese using selective culling and a high-fat diet as previously described [19].

### ALL survival experiments

ALL implantation experiments were performed on DIO and control mice at ~ 20 weeks of age. At this time, 10,000 GFP^+^ pre-B-cell ALL 8093 cells were implanted retro-orbitally. 6 or 7 days after ALL implantation, depending on the experiment, DIO mice were randomized to continue on their high-fat diet or switched to the control diet (10% calories from fat, provided ad libitum). In some experiments, monotherapy with vincristine (0.5 mg/kg/week intraperitoneal) was started on day 7, and this dose was adjusted for body weight each week. Similar experiments were performed with l-asparaginase (800 IU/kg/day, Monday–Friday) or dexamethasone (8 mg/kg/day, Monday–Friday). In other experiments, DIO and control NSG mice were implanted with LAX7 cells, and after a 17 day engraftment period, half of the DIO mice switched to control diet. On day 18, treatment with vincristine, l-asparaginase and dexamethasone (same doses as above) was started and continued for 4 weeks. Mice in all survival experiments were monitored daily for food intake and body weight, and onset of progressive leukemia (paralysis, hunched posture, palpable mass > 1 cm, poor grooming, etc.), at which time they were euthanized.

### Tissue harvesting and flow cytometry

In some experiments, mice were anesthetized with ketamine/xylazine and perfused with PBS for organ harvest at various timepoints before and after ALL implantation. When performing cell cycle analysis, mice were injected with BrdU intraperitoneally 4 h prior to organ harvest. Spleen and femurs were removed, and spleen pulp and femoral marrow were extruded with red blood cell lysis buffer (BD Biosciences, San Jose, CA, USA) for flow cytometry. Red blood cell-free bone marrow and spleen pulp were filtered through 40 μm cell strainers to create single cell suspensions. Cells utilized for cell cycle analysis were processed according to the manufacturer’s protocol (APC BrdU Flow Kit, BD Biosciences). Cells not used for cell cycle analysis were fixed in 4% PFA at 37 °C for 10 min and then permeabilized in chilled methanol for 30 min. Cells were washed and incubated in Mouse Fc Block (BD Biosciences) for 15 min then incubated for 1 h in the following fluorophore-conjugated antibodies: pEIF2α(Ser51)-PE/Cy7, pAKT(Ser473)-A647, pS6K(Thr412)-PE (Bioss USA, Boston, MA, USA). Cells were analyzed using the LSR II Flow Cytometer (BD Biosciences) in the CHLA Flow Cytometry Core. Cells were initially gated to exclude DAPI-positive dead cells and forward and side scatter to exclude debris. Gating on GFP allowed analysis of ALL vs. host cells.

### Data analysis

Kaplan Meier survival curves were generated and compared using Cox regression. Food intake was measured manually by mouse cage, and reported as daily averages or three-day moving averages as described in results. Viable cells and proportions of cells labeled with BrdU or other antibodies were compared between diet switch and DIO mice using two-sided, paired t-tests. EC_50_ was calculated by fitting normalized dose-response data to the equation: $\% viable\ cells=\frac{100}{1+{10}^{\left( Log(dose)- Log(EC50)\right)}}$. All analysis was performed using GraphPad Prism and Microsoft Excel.

## Results

### Diet restriction sensitizes ALL cells to vincristine

**Fig. 1:**
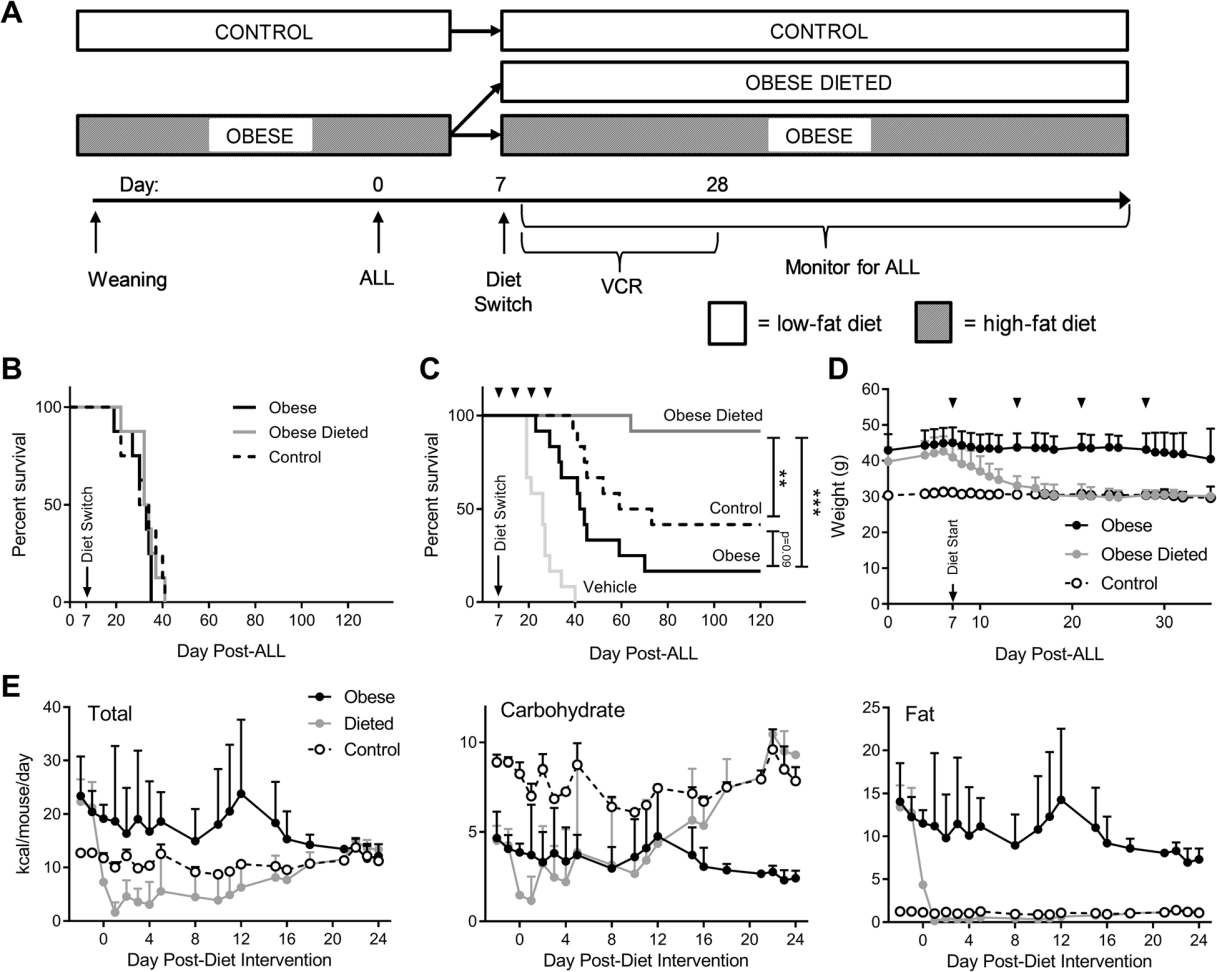
Dietary restriction improves obese leukemic C57BL/6J mouse survival after vincristine treatment. **a** Scheme showing diet intervention in survival experiments. **b** Survival of mice with ALL in each diet group with no chemotherapy treatment (*n* = 12/group). **c** Survival of mice with ALL in each diet group treated with VCR. ▼ indicate VCR doses. Vehicle-treated mice were distributed evenly between all three diet groups, and were combined due to no difference in survival. *N* = 12/group; **p < 0.01, ***p < 0.001, log rank. **d** Body weight of mice on each diet. **e** Total (left), carbohydrate (center), and fat (right) calorie daily intake in mice from each diet group. Intake is shown as daily average up until day 5 after the diet switch, and as 3 point moving average thereafter, due to high day-to-day variability. Standard deviations reflect variance between 2 and 4 cages, not individual mice. Since protein content was identical between diets, the relative intake was proportional to total caloric intake and not shown separately

To determine whether switching diet could improve ALL treatment outcome, we implanted 16 DIO and 8 non-obese control mice with syngeneic ALL cells (day 0; Fig. 1a). On day 7, half of the DIO mice were switched to the low-fat control diet, while the rest were continued on their regular diet (final *n* = 8 per group). Time to progressive ALL was not affected by diet in these mice (Fig. 1b). In other groups of mice, VCR was started on day 7, and VCR dose adjusted weekly based on body weight. In this experiment, DIO mice switched to the low-fat diet had the best survival, which was significantly better than mice maintained on the high-fat diet (*p* < 0.001), and even better than control mice that had been raised on the low-fat diet (*p* < 0.01; *n* = 6 repeated in two separate experiments for final *n* = 12 per group; Fig. 1c). Overall survival at the end of the experiment was 92% in the diet-switch group, 42% in the control group and 17% in the DIO group. Diet-switched mice in additional experiments exhibited rapid weight loss, where their body weight declined to match those of the control mice within approximately 6 days (Fig. 1d). This was associated with a substantial decline in food intake, despite access to ad libitum feed. Switched mice reduced food intake for several days after the diet switch, leading to substantially decreased intake of total calories, protein and fat (Fig. 1e). There was a relative sparing of carbohydrate intake, due to the increased carbohydrate density of the control diet. After a couple of weeks, food intake in the switched mice increased, and nutrient intake became similar to that of controls.

**Fig. 2:**
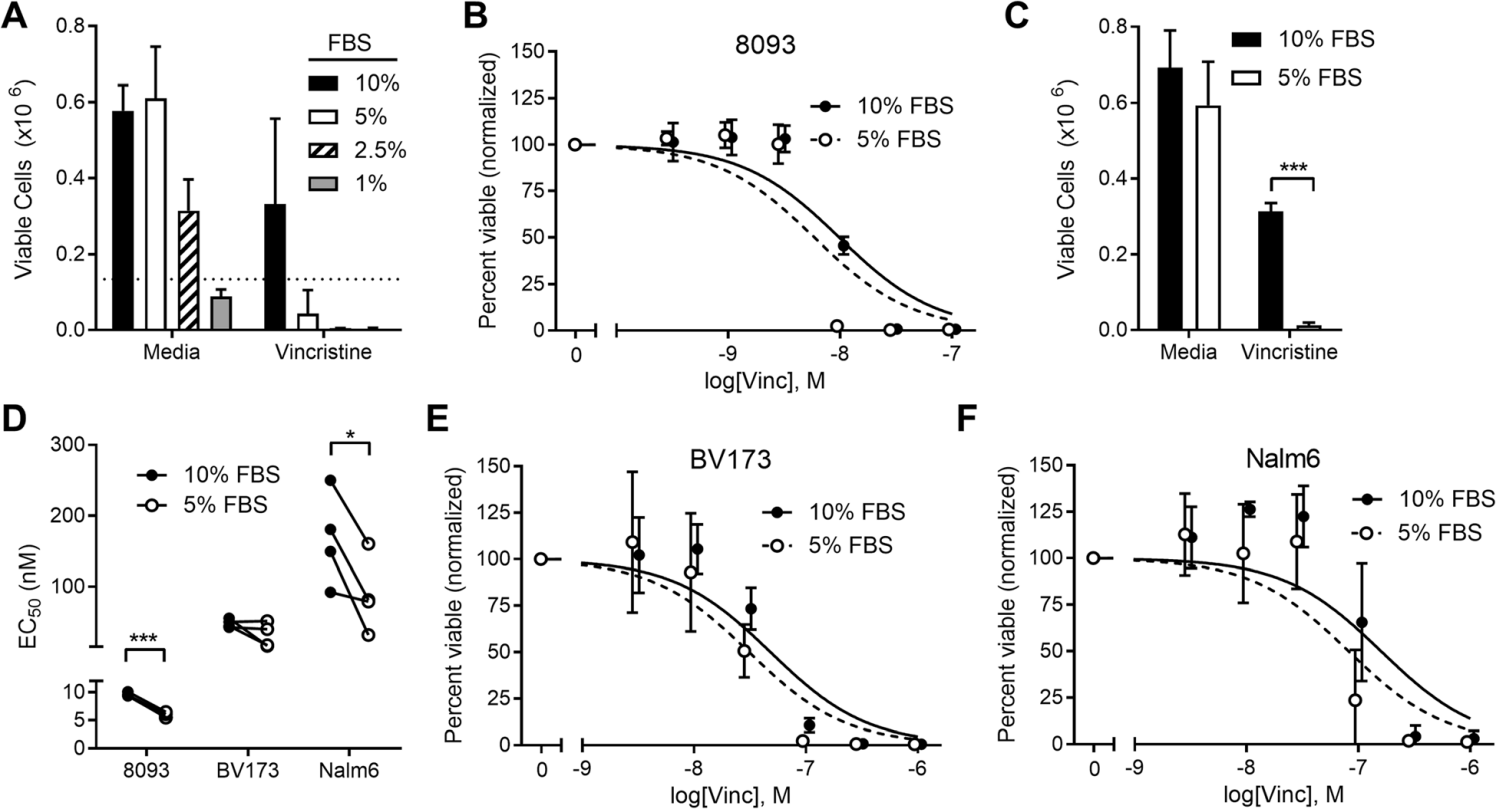
Serum restriction sensitizes ALL to vincristine treatment. **a** Viable 8093 cells after 72 h in culture media with various concentrations of FBS, alone, or with 10 nM VCR (*n* = 3). **b** Dose response of murine ALL 8093 cells cultured with VCR in 5 or 10% FBS (*n* = 4). **c** Viable 8093 cells after culture alone or with 10 nM VCR (*n* = 7) ***p < 0.001, two-sided, paired *t* test. **d** EC_50_ values calculated from dose responses of vincristine with all three cell lines; connecting lines show paired experiments. *p < 0.05, ***p < 0.001, two-sided, paired *t* test. **e**, **f** Dose response of human ALL cell lines BV173 (**e**) and Nalm6 (**f**) with VCR in 5 or 10% FBS (*n* = 4)

Dietary restriction in animals leads to a large number of hormonal, physical, physiologic, and metabolic changes that cannot be completely modeled in vitro. However, FBS restriction can simulate some of the decline in growth factors that are observed with caloric restriction and weight loss. Reducing FBS concentration increased VCR cytotoxicity (Fig. 2a); however, FBS concentrations below 5% impaired cell growth and viability. We therefore used 5% FBS, which did not affect their proliferation rate but made them significantly more sensitive to VCR (Fig. 2b, c). Similar VCR sensitization was observed in human ALL cell lines as well, BV173 and Nalm6 (Fig. 2d–f).

### Diet restriction does not alter ALL response to l-asparaginase or dexamethasone in vivo

**Fig. 3:**
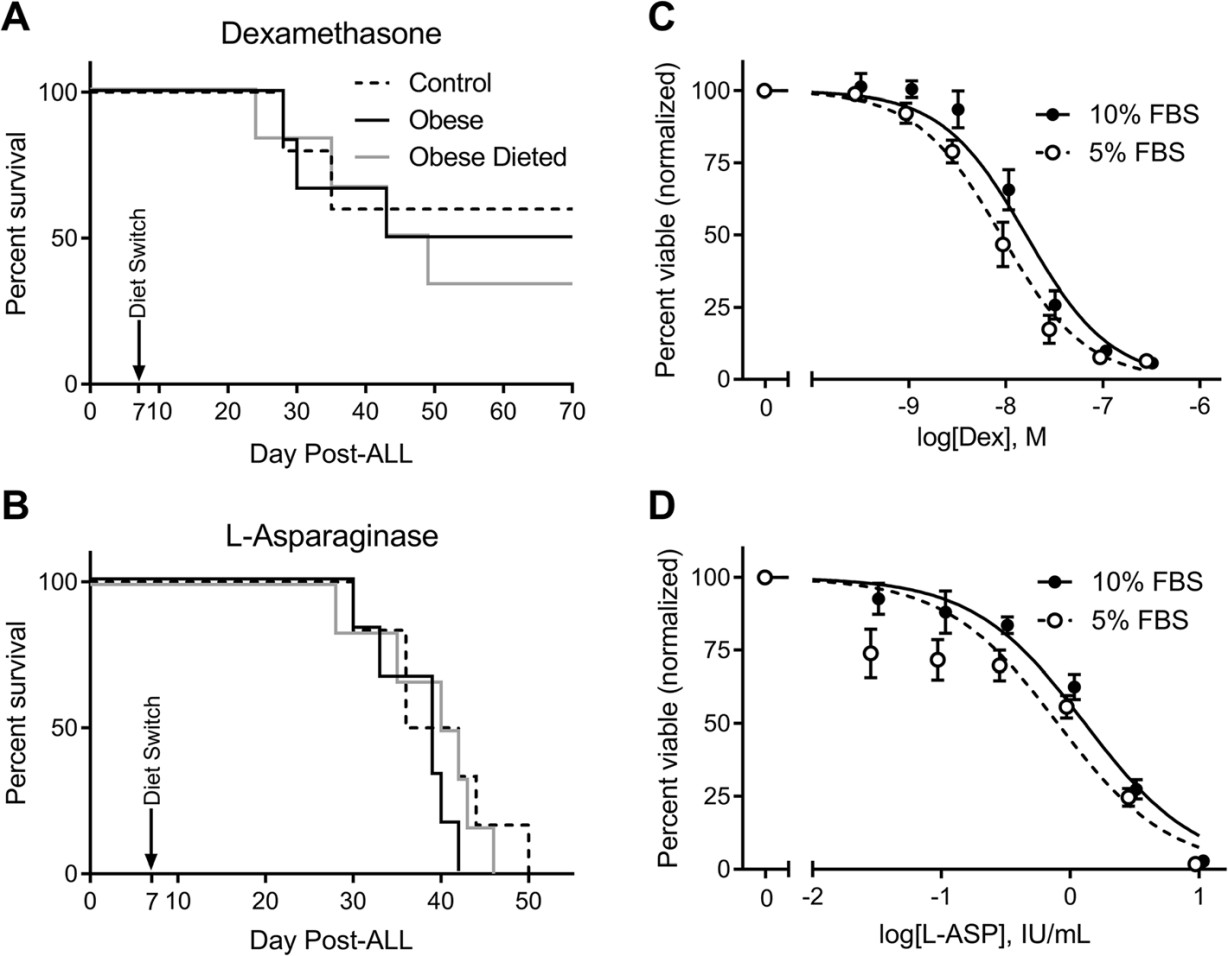
Diet restriction does not sensitize murine ALL cells to dexamethasone or l-asparaginase. **a**, **b** Survival of mice in each diet group treated with dexamethasone (**a**, *n* = 5–6) or l-asparaginase (**b**, *n* = 6). **c**, **d** Viable cells after 72 h in culture media with 5 or 10% FBS, alone or with 70 nM dexamethasone (**c**) or 2.5 IU/mL L-asparaginase (**d**), *n* = 3

Similar ALL survival experiments were done as above, but using l-asparaginase or dexamethasone monotherapy starting on day 7. Diet switch had no detectible effect on survival in these experiments (Fig. 3a, b). FBS restriction did increase 8093 cell sensitivity to dexamethasone (EC_50_ 9.1 ± 2.3 vs. 16.3 ± 3.7 nM, *p* = 0.02) and tended to increase sensitivity to l-asparaginase (EC_50_ 0.80 ± 0.22 vs. 1.30 ± 0.21 IU/mL, *p* = 0.13; Fig. 3c, d), but not to Ara-C or daunorubicin (Additional file 1: Figure S1). In addition, survival experiments performed with human ALL xenografted NSG mice treated with a combination of vincristine, l-asparaginase, and dexamethasone (VDL) showed that diet-restricted mice had no difference in survival from DIO or control mice (Additional file 2: Figure S2). Thus, the effect of dietary restriction to sensitize ALL to chemotherapy treatment in vivo appears to be relatively specific to VCR.

### Diet restriction does not alter cell cycle rate of host or ALL cells

To investigate whether dietary restriction changes cell sensitivity to VCR by altering ALL or host cell cycling rate, cells were harvested from groups of DIO mice before or after diet switch, 4 hours after BrdU injections. ALL and host cells were separated by FACS from spleens and bone marrows of DIO mice on day 6 after ALL implantation (before any diet switch), day 8 (1 day after diet switch), or day 14 (7 days after diet switch). The percentage of GFP+ ALL cells in S phase were determined and compared to GFP-host cells (splenocytes or marrow cells) from the same tissues. The percentage of cells in S phase was higher in splenic ALL cells than non-ALL cells, but similar between ALL and non-ALL cells from marrow (Additional file 3: Figure S3). Diet switch did not significantly alter this percentage in either cell type.

### Diet restriction does not alter AKT signaling in host or ALL cells

To determine how diet restriction might alter relevant intracellular signaling pathways, spleen- and marrow-derived cells from obese and obese-dieted mice (described above) were labeled with antibodies for EIF2a/pEIF2a, AKT/pAKT, and S6K/pS6K and analyzed by flow cytometry. ALL cells from both tissues showed higher phosphorylation levels of these proteins than host cells in most samples (Additional file 3: Figure S3), confirming the higher metabolic rate in cancer cells. However, diet switch had no significant effect on phosphorylation state of any of these proteins in host or ALL cells.

## Discussion

We report herein that switching obese mice from a high-fat to a low-fat diet improves ALL survival with vincristine treatment from 17 to 92%. This is the first study of which we are aware testing a diet intervention on treatment outcome from a hematological malignancy.

Other studies have evaluated whether dietary intervention can affect treatment outcome of solid cancers. Switching DIO mice from a high-fat to low-fat diet, similar to the present study, has been shown to improve survival from melanoma in mice treated with dacarbazine [20]. Ketogenic diets, containing very low carbohydrates, are used for patients with intractable epilepsy and cause decreases in blood glucose concentrations but elevations in circulating free fatty acids and ketones [21]. These diets have been shown to have an anti-tumor effect in 12 separate murine cancer models [22] and are being evaluated in patients. Fasting and short-term starvation has also been shown to improve treatment outcome from a wide variety of solid tumors in mice [12]. How efficacious these diets will be in cancer patients remains to be seen.

Our findings are consistent with retrospective data which shows that reversing obesity may be associated with improved ALL outcome in youth. Orgel et al. observed that ALL patients who had spontaneously lost weight and changed from obese to non-obese category over their treatment had better outcome than those who remained obese for > 50% of their treatment course [23]. Based on this observation, and our current findings, we have launched a clinical intervention trial of mild dietary restriction and increased physical activity in children with newly diagnosed ALL (IDEAL Weight in ALL Trial; NCT 02708108). This and similar studies are critical to help determine which cancers respond to dietary interventions and which specific interventions work for which cancers.

While we were unable to identify the mechanism whereby dietary restriction improved ALL outcome in our study, we did rule out some important mechanisms. VCR cytotoxicity is cell cycle dependent, as opposed to l-asparaginase and dexamethasone, which could explain why mice treated with dexamethasone or l-asparaginase derived no survival benefit from a dietary switch. However, we did not detect an effect of dietary intervention on cell cycle of ALL or host cells one or 7 days after diet initiation. We also found no effect of diet intervention on the ALL or host cell activity of pathways commonly evaluated for effects of dietary intervention on cancer, though we did observe an increased activity of these pathways in the cancer cells vs. host cells. Lack of effect of diet on AKT or S6K phosphorylation implies that our intervention was not acting through IGF-1 or mTOR, respectively. eIF2a phosphorylation is observed with amino acid deprivation, oxidative stress, and unfolded protein response [24], arguing against these potential mechanisms of our dietary intervention. It is possible that these signals may have mediated effects after 7 days, which we would have missed with our experimental design. Further, while these findings argue against these pathways, we did not exhaustively rule them out, which would require extensive additional experiments and should be evaluated in future studies.

It is also possible that the protective effect of a dietary switch is mediated in part by immunological mechanisms. In the 2010 update to the well-known “Hallmarks of Cancer,” four new principles were identified as vital to carcinogenesis and cancer cell survival, one being evasion of immune destruction [25]. In addition to their direct cytotoxic effect, some chemotherapies have been identified as inducers of immunogenic cell death, whereby their mechanism of cytotoxicity invokes a host T-cell-mediated immunologic response [26]. These include the anti-microtubule agents docetaxel and paclitaxel, as well as the anthracyclines, daunorubicin and doxorubicin, and the anthracendione, mitoxantrone. Interestingly, caloric restriction and short-term fasting have been shown to improve T-cell anti-tumor reactivity and reduce immune-suppressing T-regs. Combined, these studies suggest that dietary restriction could synergize with a certain subset of chemotherapies to augment host anti-tumor response and improve treatment outcomes. This would be consistent with our finding that NSG mice, which lack immune systems, obtained no benefit from the dietary switch. On another note, it is particularly interesting that three of the other four identified immune inducers, daunorubicin, doxorubicin, and mitoxantrone, are commonly used in primary and relapse ALL chemotherapy regimens. These chemotherapeutic agents were not studied in our current model but should be tested in future studies.

Our study has several limitations. Perhaps our biggest limitation is that we did not detect an effect of dietary intervention in our human xenograft model. This limits the degree to which our results may be directly translatable to human leukemia. The lack of effect in this model could have been due to the triple chemotherapy regimen used, which could have masked a small effect of vincristine alone, to the immunodeficient state as discussed above, or to other mouse strain differences. In any case, dietary intervention in human xenograft models should be further evaluated so that relevance to human leukemia can be confirmed. Another limitation is that we tested a relatively crude dietary intervention—switching DIO mice from a high-calorie/high-fat diet to a lower-calorie/low-fat diet. This switch induced several changes, including decreased calorie, protein, and fat intake, while sparing carbohydrate intake. These changes undoubtedly altered the animals’ insulin sensitivity, circulating growth factors, body composition, and fuel availability. Indeed, another study which utilized a similar high-fat to low-fat diet switch in C57Bl/6 mice showed a number of physiological effects, including decreases in glucose and insulin, improvement in lipids and free fatty acids, and decrease in several pro-inflammatory cytokines and adipokines [20]. This broad range of effects may contribute to the efficacy of this intervention, but at the same time makes identification of specific mechanisms difficult. Thus, further study will need to be done to determine which of these changes and effects contribute to the improved survival. On the other hand, murine obesity induced by a high-fat diet is not completely analogous to human obesity, which is generally associated with excess carbohydrate intake; thus, utility of these models for this purpose may be limited. Finally, despite the large observed effect of dietary intervention on vincristine efficacy, the mechanism behind this effect remains elusive.

While treatment of childhood ALL has led to substantial improvements in survival, there are still ~ 8% children who relapse from this disease every year [27]. In addition, children with certain ALL subtypes, adolescents and adults, and obese patients are all at increased risk of relapse and mortality. Given the substantial toxicity of current chemotherapy regimens, it is important to investigate alternative approaches to improve ALL outcome without adding additional chemotherapy agents.

## Conclusions

Switching obese mice with ALL from a high-fat to low-fat diet substantially improved survival with VCR treatment, though not with l-asparaginase or dexamethasone treatment. The finding that a dietary intervention can improve ALL treatment outcome in a preclinical model should be further studied so that its potential benefit can be evaluated in both obese and non-obese patients.

## Additional files

Additional file 1: **Figure S1.** Serum reduction does not improve efficacy of Ara-C or DNR in vitro. A–C Viable 8093 (A), BV173 (B), and Nalm6 (C) cells after 72 h exposure to chemotherapy Ara-C (left) or DNR (right) *N* = 4. (TIF 979 kb)

Additional file 2: **Figure S2. **Dietary restriction does not affect ALL outcome in xenograft NSG model treated with vincristine, dexamethasone, and l-asparaginase (VDL). Obese-dieted *n* = 8, obese *n* = 6, control *n* = 5. (TIF 183 kb)

Additional file 3: **Figure S3.** Effect of dietary restriction on host and ALL cell cycle and Akt pathway activation. A Percentage of BrdU+ non-ALL and ALL cells from spleens (left) and marrow (right) of DIO mice after ALL implantation on day 6 (1 day before diet switch), day 8 (1 day after diet switch), and day 14 (7 days after diet switch; *n* = 3). B–D Phosphoprotein levels from flow cytometry of pS6K (B), pAKT (C), and pEIF2α (D) from cells described in A. (TIF 130 kb)

## Abbreviations

ALL: Acute lymphoblastic leukemia
DIO: Diet induced obesity
DNR: Daunorubicin
EC_50_: Effective concentration that kills 50% of the cells
FBS: Fetal bovine serum
MRD: Minimal residual disease
NSG: NOD/SCID IL2R gamma chain knockout
VCR: Vincristine
