# Supplementary figures and images for: Switch to low-fat diet improves outcome of acute lymphoblastic leukemia in obese mice
Source: Cancer Metab. 2018 Nov 1;6:15. doi: 10.1186/s40170-018-0189-0 (PMC6211598; doi:10.1186/s40170-018-0189-0)

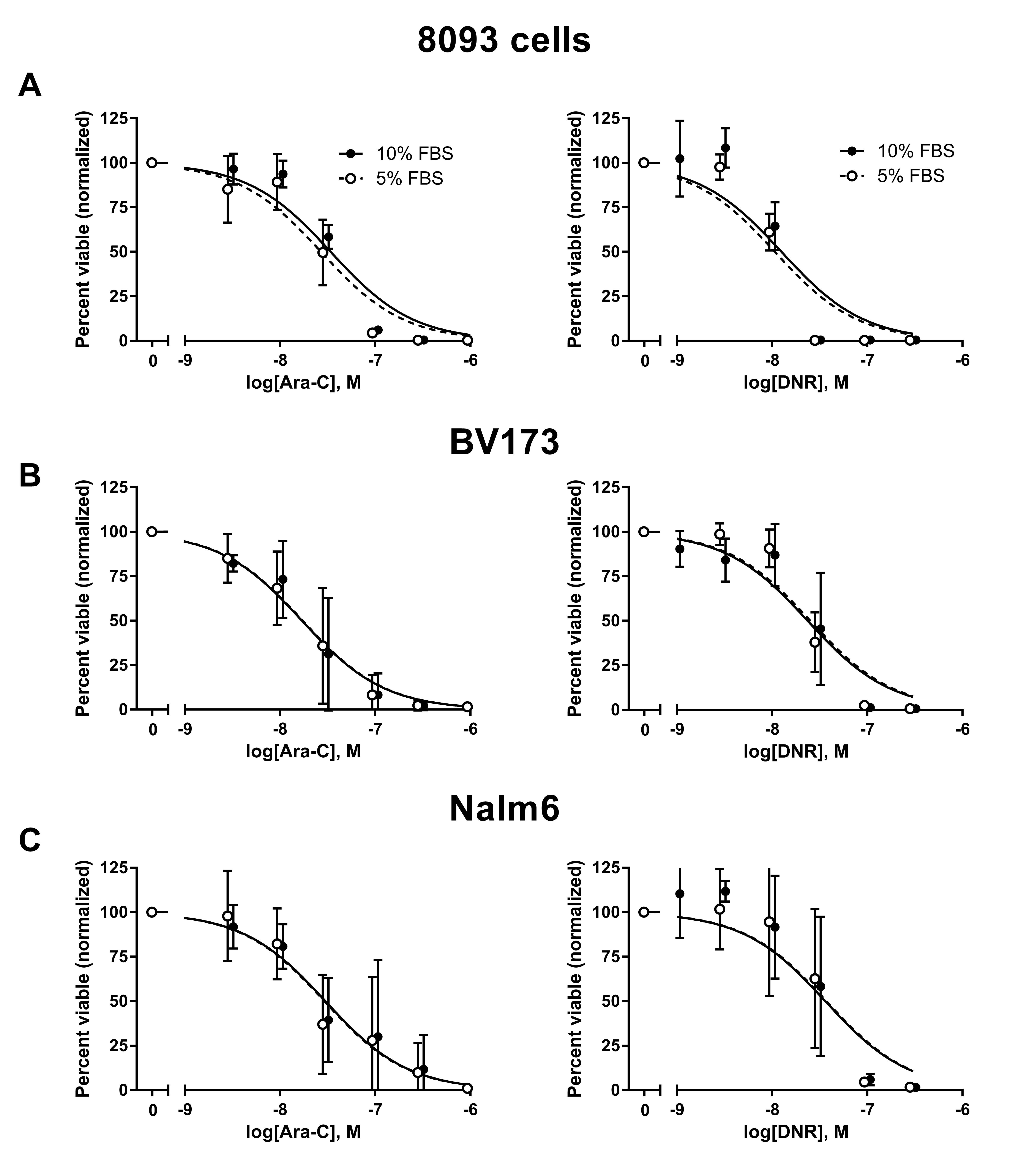

Supplement: Supplementary file 1 — Figure S1. Serum reduction does not improve efficacy of Ara-C or DNR in vitro. A–C Viable 8093 (A), BV173 (B), and Nalm6 (C) cells after 72 h exposure to chemotherapy Ara-C (left) or DNR (right) N = 4. (TIF 979 kb) [file 40170_2018_189_MOESM1_ESM.tif]

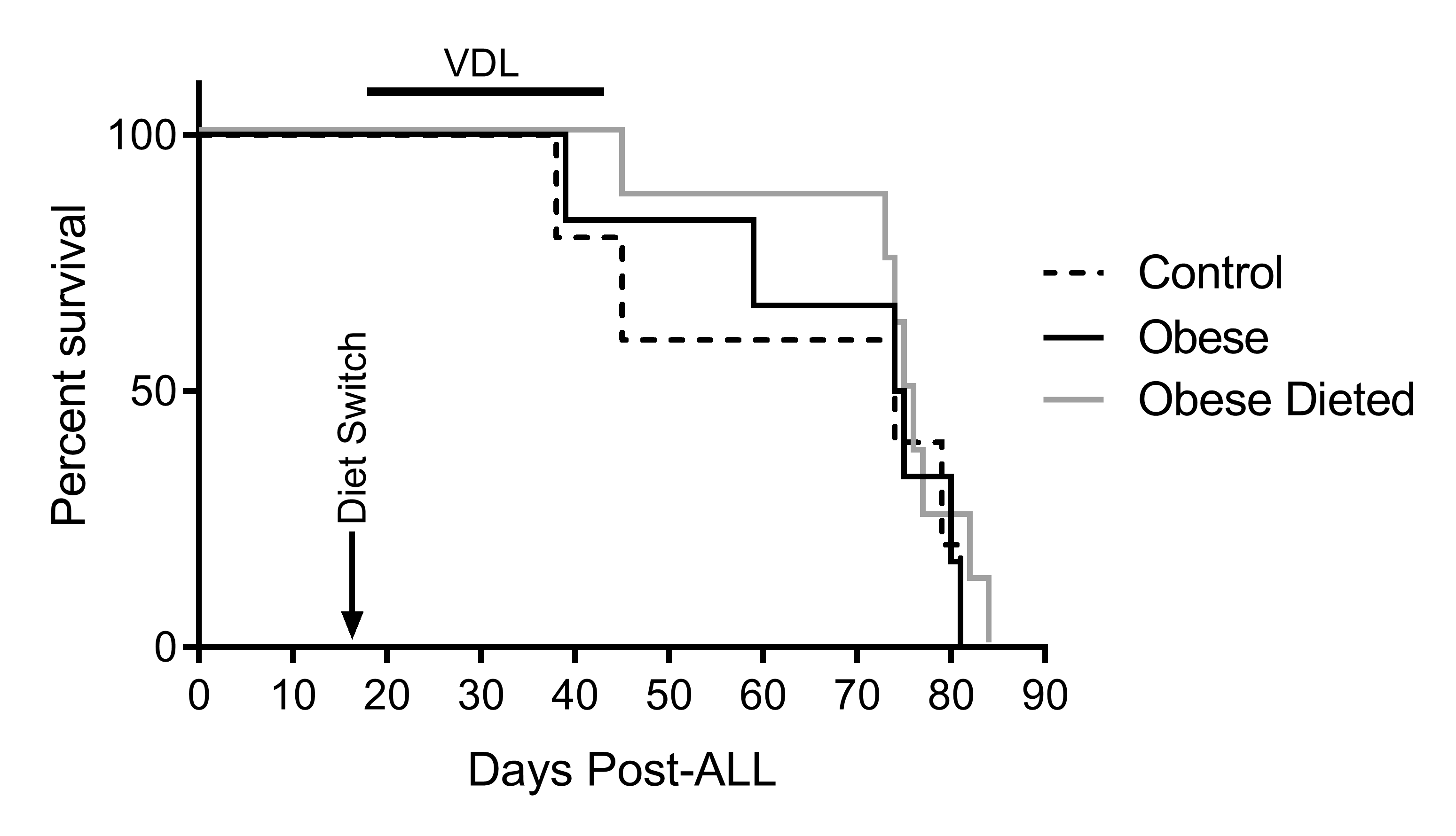

Supplement: Supplementary file 2 — Figure S2. Dietary restriction does not affect ALL outcome in xenograft NSG model treated with vincristine, dexamethasone, and l-asparaginase (VDL). Obese-dieted n = 8, obese n = 6, control n = 5. (TIF 183 kb) [file 40170_2018_189_MOESM2_ESM.tif]

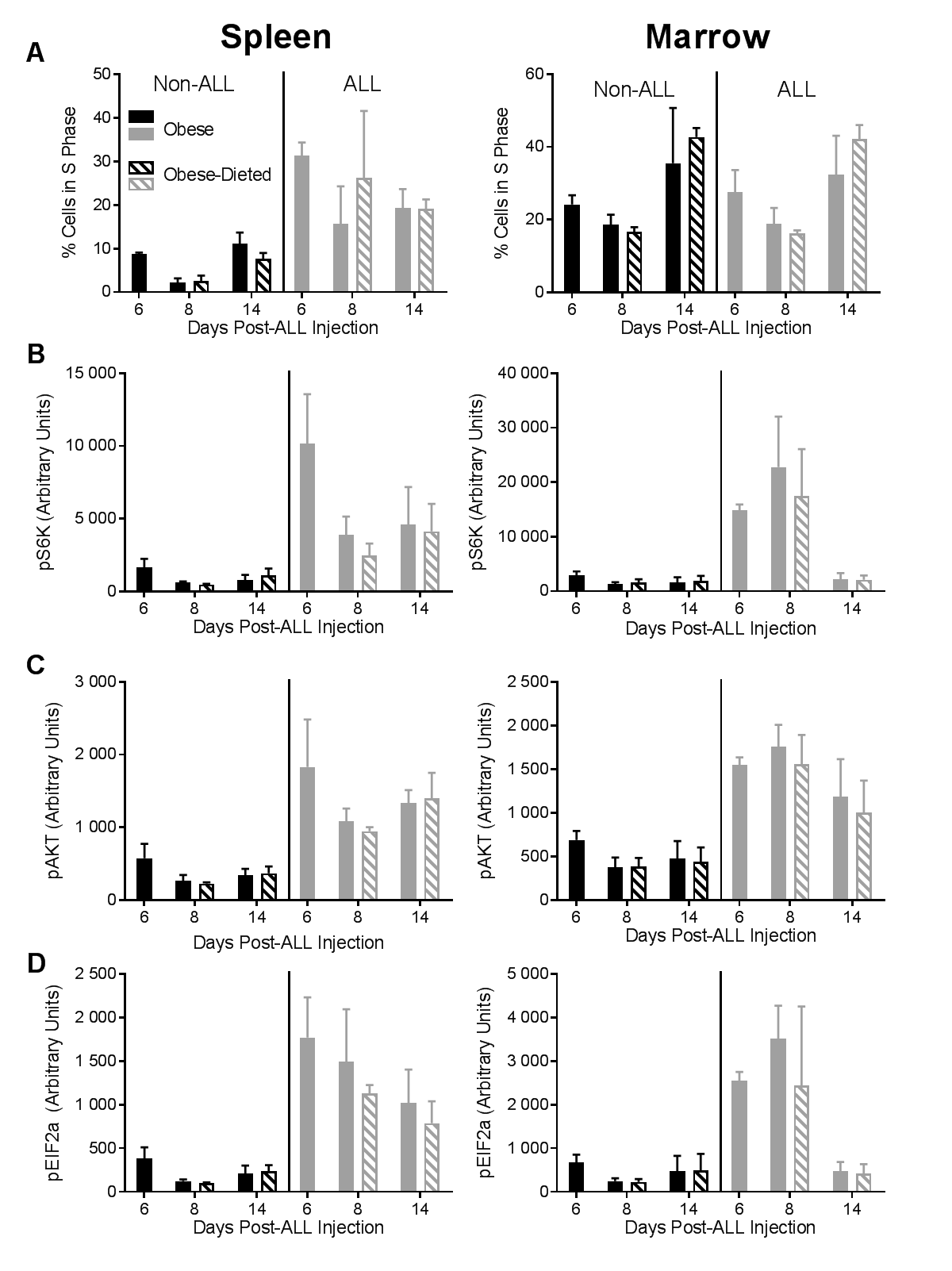

Supplement: Supplementary file 3 — Figure S3. Effect of dietary restriction on host and ALL cell cycle and Akt pathway activation. A Percentage of BrdU+ non-ALL and ALL cells from spleens (left) and marrow (right) of DIO mice after ALL implantation on day 6 (1 day before diet switch), day 8 (1 day after diet switch), and day 14 (7 days after diet switch; n = 3). B–D Phosphoprotein levels from flow cytometry of pS6K (B), pAKT (C), and pEIF2α (D) from cells described in A. (TIF 130 kb) [file 40170_2018_189_MOESM3_ESM.tif]
